# How medical insurance payment systems affect the physicians’ provision behavior in China—based on experimental economics

**DOI:** 10.3389/fpubh.2024.1323090

**Published:** 2024-05-02

**Authors:** Shuyan Lin, Qiang Sun, Hezeng Zhou, Jia Yin, Chao Zheng

**Affiliations:** ^1^Center for Health Management and Policy Research, School of Public Health, Cheeloo College of Medicine, Shandong University, Jinan, China; ^2^NHC Key Lab of Health Economics and Policy Research (Shandong University), Jinan, China; ^3^Zhucheng Mengtuan Hospital, Zhucheng, China

**Keywords:** payment systems, fee-for-service, diagnosis-intervention package, physicians’ provision behavior, China

## Abstract

**Background:**

It introduced an artefactual field experiment to analyze the influence of incentives from fee-for-service (FFS) and diagnosis-intervention package (DIP) payments on physicians’ provision of medical services.

**Methods:**

This study recruited 32 physicians from a national pilot city in China and utilized an artefactual field experiment to examine medical services provided to patients with different health status.

**Results:**

In general, the average quantities of medical services provided by physicians under the FFS payment were higher than the optimal quantities, the difference was statistically significant. While the average quantities of medical services provided by physicians under the DIP payment were very close to the optimal quantities, the difference was not statistically significant. Physicians provided 24.49, 14.31 and 5.68% more medical services to patients with good, moderate and bad health status under the FFS payment than under the DIP payment. Patients with good, moderate and bad health status experienced corresponding losses of 5.70, 8.10 and 9.42% in benefits respectively under the DIP payment, the corresponding reductions in profits for physicians were 10.85, 20.85 and 35.51%.

**Conclusion:**

It found patients are overserved under the FFS payment, but patients in bad health status can receive more adequate treatment. Physicians’ provision behavior can be regulated to a certain extent under the DIP payment and the DIP payment is suitable for the treatment of patients in relatively good health status. Doctors sometimes have violations under DIP payment, such as inadequate service and so on. Therefore, it is necessary to innovate the supervision of physicians’ provision behavior under the DIP payment. It showed both medical insurance payment systems and patients with difference health status can influence physicians’ provision behavior.

## 1 Introduction

Medical insurance departments employ various insurance payment systems to settle expenses with medical institutions. In China, the fee-for-service (FFS) payment has been used for a long time. However, under the FFS payment, some physicians provide unnecessary medical services to increase their income, resulting in a significant rise of medical costs, conflicts between doctors and patients, and the increasingly significant problem of “difficult and expensive access to medical care” (1). In response to these challenges, China has implemented a series of reforms to medical insurance payment systems. In 2020, the Chinese government implemented the diagnosis-intervention package (DIP) payment, which is an innovative medical insurance payment method based on point calculation under the regional budget. The DIP payment is a packing payment model based on disease characteristics and making full use of the advantages of medical data and innovation of big data technology. It can accurately adapt the cost and determine the price with little manual intervention, which can reply to the issues of price discovery in the reform of medical insurance payment. The DIP payment system establishes a system of relative prices for different diseases, with fixed scores for each DIP disease group to reflect the relative level of resource dissipation for treating the disease. In the medical insurance payment link, the medical insurance operator makes advance payment in accordance with 95% of the DIP coordinated fund hospitalization recorded costs declared by each designated medical institution in each month, and the annual liquidation is carried out in each natural year, and the medical insurance operator calculates the unit price of points based on the total amount of the city’s annual medical insurance fund divided by the city’s total number of points in the medical institutions, which in turn leads to the total amount of payment. While the relative prices for different DIP disease groups are fixed, the actual prices are determined *a posteriori* on the basis of the price per unit of points.

Because the effectiveness of medical insurance payment system reform primarily depends on the responses of medical service providers, scholars have carried out related researches. Previous research have shown that the DIP payment improves the efficiency of medical services, optimizes the structure of medical costs and reduces the waste of medical resources. Some scholars adopt the difference-in-difference method and found the DIP payment reform achieve a short-term success in slowing down the growth of health expenditures (2). Since 2018, except Shenzhen, Guangdong has promoted the reform of DIP payment, and made certain achievements in cost control, cost reduction, quality assurance and efficiency improvement (3). The study found that on the whole, the reform areas of the DIP payment show a trend of slower growth of medical insurance fund expenditure, slower growth of hospitalization expenses, lower proportion of drug consumption, lower average length of stay in hospital, and lower *per capita* conceit ratio (4).

Academics have found that the DIP payment can regulate doctors’ behavior. Qian et al. (5) investigated the largest DIP pilot city of China and found that the DIP-based payment help regulate provider behaviors when treating high-risk patients. A study found that the DIP payment incentivizes physicians to provide more than the optimal quantities for mild and moderate patients, but there was less over-provision under DIP than under FFS (6). Some studies have also documented that doctors sometimes have violations under DIP payment such as cost shifting, decomposed hospitalization, and surgical upgrading (7, 8). The study also showed that expenditures per admission generally decrease after the introduction of the DIP payment, but unintended consequences such as unnecessary admissions, early discharge, and up-coding emerge (2). Therefore, it is important to study the effect of DIP payment on physicians’ behavior.

Recent years, the use of experimental economics in the medical service has grown and experimental economics is mainly used to study the behavior of medical service providers. Experimental economics refers to the experimental methods in economics, including the laboratory experiment and the field experiment, which mainly translates the rules into the environment required for experiment, carries out repeated tests and comparisons of an economic phenomenon in order to improve the theory and provide people with a basis for decision making (9). In 2011, Hennig-Schmidt et al. (10) initiated a laboratory experiment to investigate the influence of incentives from fee-for-service and capitation (CAP) payments on physicians’ provision of medical services. Brosig-Koch et al. (11) also conducted artefactual field and laboratory experiments to examine how physicians, medical students, and non-medical students respond to financial incentives from FFS and CAP systems. Godager et al. (12) used date in 2011 from a laboratory experiment by Hennig-Schmidt et al. (10) to investigate physician altruism and found that physicians are highly concerned about the health outcomes of their patients. Green (13) utilized a real-effort laboratory experiment to study the impact of fee-for-service, capitation, salary, and pay-for-performance on physicians’ behavior. Some related studies have employed laboratory experiments in China, albeit in limited numbers. For instance, Zhang et al. (14) used a laboratory experiment and recruited 120 students as subjects to investigate the impact of incentives from fee-for-service and diagnosis-related group (DRG) payments on physicians’ supply of medical services. Tan et al. (15) used medical students as doctors to make medical decisions under FFS and DIP payments and found physicians’ provision behavior can be effectively regulated under DIP payment.

Laboratory experiment refers to carrying out experiment in the laboratory, selecting college students as investigation objects and letting them play a certain role to make corresponding decisions. Field experiments are carried out in real social environments. Consequently, laboratory experiments possess good internal validity but lack external validity. On the other hand, field experiments are closer to real-world conditions and exhibit better external validity compared to laboratory experiments (16). Therefore, this study creatively used an artefactual field experiment to investigate the impact of FFS and DIP payments on physicians’ behavior in the real world. On the one hand, this study made up for the deficiency of laboratory experiment and obtained high quality research evidence. On the other hand, it measured and compared patient benefit and personal net profit generated by doctors’ provision behavior under different payment methods in the real world.

## 2 Materials and methods

### 2.1 Study setting and object

#### 2.1.1 Study setting

In 2020, Dongying City initiated the DIP payment, becoming the national pilot city in China. Dongying City has utilized the DIP payment to compensate local tertiary and secondary hospitals for their services. So considering the geographical location, economic level and feasibility, this study choosen Dongying City as the research area. A level A of tertiary general hospital and a level A of secondary general hospital were selected as the research sites in Dongying City.

#### 2.1.2 Study object

The offline questionnaire surveys were conducted among doctors in a level A of tertiary general hospital and a level A of secondary general hospital. Physicians were required to meet the following inclusion criteria. First, physicians worked at least three years. Second, physicians experienced the FFS and DIP payments. Third, physicians worked properly. Clinicians who were unwilling to participate in this survey were excluded.

### 2.2 Study method

#### 2.2.1 Questionnaire content

This study used the questionnaire survey. Based on domestic and foreign literatures, the questionnaire was formed. The questionnaire included the content of artefactual field experiment under FFS and DIP payments.

The experimental content was represented in the form of table. The first two columns displayed medical services and their corresponding quantities. Column three contained the physicians’ payments, while column four displayed the costs of medical services. Column five represented physicians’ profits (payments subtracted costs), while column six indicated patients’ benefit. Different numbers of medical services were associated with varying physicians’ payments, costs, physicians’ profits, and patients’ benefit.

##### 2.2.1.1 Experimental design

Many literatures using experimental economics all classified the three health status of patients in experiments as good, moderate and bad (10–15). So, this experiment utilized five abstract illnesses (*k* = A, B, C, D, and E) and three patient health status which were good, moderate, and bad (*j* = 1, 2, and 3). Each physician was required to determine the quantities of medical services (0 to 10) to offer to 15 different patient types within each payment. Therefore, every physician needed to make 30 decisions totally.

Tokens were utilized as units in the experiment, and the physicians’ decisions determined their profits and patients’ benefits. Upon completing all decisions, physicians were paid the sum of profits corresponding to 30 decisions at a rate of 10 tokens = 1 RMB. Simultaneously, patient benefits corresponding to 30 decisions were converted into money at a rate of 10 tokens = 1 RMB and donated to a real patient, donation details would be emailed to the physicians for monitoring purposes.

##### 2.2.1.2 Experimental parameters

This study established the experimental parameters based on the characteristics of each payment. The consultation fee was denoted as *R_jk_* (*q*), the cost as *C_jk_* (*q*), the physician’s profit as π_jk_ (*q*), and the patient benefit as *B_jk_* (*q*).

In the FFS payment, the consultation fee increased as the quantity of medical services increased. The specific payment amount varied depending on the illnesses. The payment parameters in this study were derived from the experimental parameters utilized by Hennig-Schmidt’s study (10), which utilized the German scale of charges and fees for physician services (EBM). By comparing the prices of 0–10 unit service items in a hospital of Beijing with the experimental parameters in Hennig-Schmidt’s study (10), Zhang et al. (14) found there were no statistical significance in setting the consultation fee for FFS between China and Germany. Fees were highest (16.60, 22.50, 18.30, 23.60, 23.00) under FFS when doctors provided 10 services for patients with the types of diseases (A, B, C, D, E). Tan et al. (15) adopted a laboratory experiment, selected college students as investigation objects and let them play the role of doctors in the experiment to make medical decisions under DIP payment. Therefore, the parameters of doctors’ treatment fees under DIP payment in the real world referenced the experimental parameters of Tan et al. (15), which were related to the type and severity of diseases. The payment remained the same regardless of the quantity of medical services but varied depending on the illness. Patients gained the optimal benefits when physicians provided 3, 5 and 7 medical services respectively to patients with the health status of good, moderate and bad under both payments. Therefore, the optimal quantities were q^*^_1k_ = 3, q^*^_2k_ = 5, and q^*^_3k_ = 7 for patients with the health status of good, moderate and bad. Used the optimal quantities as the benchmarks to identify underservice and overservice. The general optimal medical service quantities were q^*^_jk_ = 5 [(q^*^_1k_ + q^*^_2k_ + q^*^_3k_) /3].

The cost parameters employed the convex cost function assumed by Ma (17). Physicians incurred costs according to *C_jk_* (*q*) = 0.1 × *q*^2^ in both conditions, which were independent of payment systems, the type and severity of diseases. Costs were greatest when doctors provided 10 services to patients under both payments.

The parameters for physicians’ profits were calculated as the consultation fees subtracted costs. Profits varied among illnesses in both FFS and DIP payments due to changes in payment amounts while costs remained constant. Physicians got the maximum profits (8.00) when the amount of medical services provided to patients the types of disease (A) were 5. Moreover, Physicians gained the highest profits (12.50, 8.30, 13.60, 13.00) when they provided 10 services for patients with the types of diseases (B, C, D, E). Therefore, the general maximum profits under FFS were 11.08 [(8.00 × 3 + 12.50 × 3 + 8.30 × 3 + 13.60 × 3 + 13.00 × 3)/15]. While doctors got the highest profits (10.00, 13.00, 9.00, 15.00, 12.00) under DIP when they provided 0 medical services for patients with the types of diseases (A, B, C, D, E). So the general maximum profits under DIP were 11.80 [(10.00 × 3 + 13.00 × 3 + 9.00 × 3 + 15.00 × 3 + 12.00 × 3)/15].

The experimental parameters for patient benefits were derived from the study conducted by Hennig-Schmidt et al. (10). Patient benefits varied based on their health states. Patients with the health status of good, moderate and bad achieved the corresponding optimal benefits (10.00, 10.00, 9.45) when physicians provided 3, 5, and 7 medical services. Therefore, the general best benefits for patients under FFS and DIP payments were 9.82 [(9.45 × 5 + 10.00 × 5 + 10.00 × 5)/15]. The specific experimental parameters were presented in Table 1.

**Table 1:** Experimental parameters.

Payment	Variable	Quantity										
		0	1	2	3	4	5	6	7	8	9	10
FFS	R_jA_ (q)	0.00	1.70	3.40	5.10	5.80	10.50	11.00	12.10	13.50	14.90	16.60
	R_jB_ (q)	0.00	1.00	2.40	3.50	8.00	8.40	9.40	16.00	18.00	20.00	22.50
	R_jC_ (q)	0.00	1.80	3.60	5.40	7.20	9.00	10.80	12.60	14.40	16.20	18.30
	R_jD_ (q)	0.00	2.00	4.00	6.00	8.00	8.20	15.00	16.90	18.90	21.30	23.60
	R_jE_ (q)	0.00	1.00	2.00	6.00	6.70	7.60	11.00	12.30	18.00	20.50	23.00
DIP	R_jA_ (q)	10.00	10.00	10.00	10.00	10.00	10.00	10.00	10.00	10.00	10.00	10.00
	R_jB_ (q)	13.00	13.00	13.00	13.00	13.00	13.00	13.00	13.00	13.00	13.00	13.00
	R_jC_ (q)	9.00	9.00	9.00	9.00	9.00	9.00	9.00	9.00	9.00	9.00	9.00
	R_jD_ (q)	15.00	15.00	15.00	15.00	15.00	15.00	15.00	15.00	15.00	15.00	15.00
	R_jD_ (q)	12.00	12.00	12.00	12.00	12.00	12.00	12.00	12.00	12.00	12.00	12.00
FFS	π_jA_ (q)	0.00	1.60	3.00	4.20	4.20	8.00	7.40	7.20	7.10	6.80	6.60
	π_jB_ (q)	0.00	0.90	2.00	2.60	6.40	5.90	5.80	11.10	11.60	11.90	12.50
	π_jC_ (q)	0.00	1.70	3.20	4.50	5.60	6.50	7.20	7.70	8.00	8.10	8.30
	π_jD_ (q)	0.00	1.90	3.60	5.10	6.40	5.70	11.40	12.00	12.50	13.20	13.60
	π_jE_ (q)	0.00	0.90	1.60	5.10	5.10	5.10	7.40	7.40	11.60	12.40	13.00
DIP	π_jA_ (q)	10.00	9.90	9.60	9.10	8.40	7.50	6.40	5.10	3.60	1.90	0.00
	π_jB_ (q)	13.00	12.90	12.60	12.10	11.40	10.50	9.40	8.10	6.60	4.90	3.00
	π_jC_ (q)	9.00	8.90	8.60	8.10	7.40	6.50	5.40	4.10	2.60	0.90	−1.00
	π_jD_ (q)	15.00	14.90	14.60	14.10	13.40	12.50	11.40	10.10	8.60	6.90	5.00
	π_jE_ (q)	12.00	11.90	11.60	11.10	10.40	9.50	8.40	7.10	5.60	3.90	2.00
FFS DIP	C_jk_ (q)	0.00	0.10	0.40	0.90	1.60	2.50	3.60	4.90	6.40	8.10	10.00
	B_1k_ (q)	0.00	1.00	1.50	10.00	9.50	9.00	8.50	8.00	7.50	7.00	6.50
	B_2k_ (q)	0.00	0.75	1.50	2.00	7.00	10.00	9.50	9.00	8.50	8.00	7.50
	B_3k_ (q)	0.00	0.75	2.20	4.05	6.00	7.75	9.00	9.45	8.80	6.75	3.00

#### 2.2.2 Questionnaire collection

In March 2023, the researchers went to Dongying to carry out the field investigations in a level A of tertiary general hospital and a level A of secondary general hospital.

Before doctors began to fill out the questionnaires, the trained investigators explained the design of the experiment and the questionnaires also included the description of the experiment. Doctors must correctly answer the relevant comprehension questions on the questionnaires before they began to fill in the questionnaires. If doctors had any questions during the questionnaires filling, they could ask the investigators in time and were forbidden to discuss the answers with other doctors. Doctors who violate the rules would not get experimental pay. After a doctor filled in the questionnaire, the investigator firstly checked whether the questionnaire was complete and whether the amount of medical services was within the range of 0–10. If there was any missing filling or vague answers, the investigator checked with the doctor on the spot. Secondly, The investigator calculated the sum of the doctor’s net profit under the two payment systems. Finally, the investigator transferred the money to the doctor on the spot according to the ratio of 10 tokens =1 RMB.

#### 2.2.3 Inclusion and exclusion criteria of the questionnaires

In order to ensure the consistency of the questionnaires completed by survey subjects and more in line with the reality questionnaires should have met the following inclusion criteria. First, the questionnaires were filled out completely. Second, physicians provided more medical services to severe patients than moderate patients. Third, physicians provided more medical services to moderate patients than mild patients. The exclusion criteria for the questionnaires were as follows. Firstly, the amounts of medical services that doctors provide to patients were not between 0 and 10 under two payment methods. Secondly, the doctor discussed the questionnaire answers with others.

A total of 32 questionnaires were sent out and 32 were recovered. After strict screening, 32 questionnaires met the inclusion criteria and there were 16 questionnaires respectively from a level A of tertiary general hospital and a level A of secondary general hospital.

### 2.3 Statistical analysis

This study analyses four aspects which were the quantity of medical services, patients benefit, physicians’ profit and decisions relating to the optimal quantity of care. Since the data was not follow a normal distribution, the Wilcoxon signed-rank test and the Mann–Whitney U test were employed to analyze the numerical variable data. The Pearson chi-square test was used to analyze categorical variables.

### 2.4 Quality control

There were corresponding quality control methods in the research design stage, data collection stage and data sorting stage. Based on the domestic and foreign literatures, the questionnaire was formed. Before data collection, the investigators were uniformly trained on experimental purpose, experimental design, calculation of experimental compensation, and matters needing attention in the investigation. Other quality control measures during the data collection phase could be seen in “Questionnaire collection.” The collected questionnaires were sorted out and screened according to the inclusion and exclusion criteria of the questionnaires in data collation stage. After the screening, one person was responsible for inputting questionnaires data that met the inclusion criteria, and one person was responsible for verifying the inputting results.

## 3 Results

### 3.1 Quantity of medical services

First, physicians were found to provide 12.88% more medical services under the FFS payment compared to the DIP payment. The result of the Mann–Whitney U test indicated both payment systems existed the significant difference and physicians under the FFS payment provided significantly more medical services than under the DIP payment.

The average quantities of medical services provided by physicians under the FFS payment were 5.52, and the mean deviations between the real quantities and the optimal quantities were 0.52, which was statistically significant. These results indicated that patients were overserved under the FFS payment. While the average quantities of healthcare services provided by physicians under the DIP payment were 4.89 and the deviations from the optimal quantities were-0.11, which was not statistically significant.

Secondly, physicians provided 24.49, 14.31, and 5.68% more medical services to patients with the health status of good, intermediate, and bad under the FFS payment than under the DIP payment. The behavioral differences between the two payment systems existed when comparing physicians’ quantity choices to patients with three health status. These meant the gap in the amount of medical services would narrow when patients’ health status deteriorated.

Under the FFS payment, the average quantities of medical services provided for patients with the health status of good and intermediate were 4.27 and 5.59, respectively. The average quantities were 42.33 and 11.80% higher than the corresponding optimal quantities and the differences were statistically significant. The average quantities of medical services received by patients with bad health status were 6.70, which were 4.29% less than the corresponding optimal quantities, and the difference was not statistically significant. In summary, as the demand for medical services increased, the level of oversupplies decreased. This indicated that the FFS payment is more beneficial for patients with relatively poor health status.

Under the DIP payment, the average quantities of medical services received by patients with the health status of good and bad were 3.43 and 6.34. These values differed from the corresponding optimal quantity by 14.33 and-9.43%, the both differences were statistically significant. Patients with moderate health status received 2.20% less medical services than the optimal quantities, and the difference was not statistically significant. Thus, the DIP payment is more favorable for patients with moderate health status. The detailed findings could be found in Table 2.

**Table 2:** Physicians’ quantity choices for patients with different health status under the FFS and DIP payments.

Variable	Mean	Median	SD	Comparison with the optimal quantities (P)
FFS	5.52	5.27	1.21	0.045
Good	4.27	3.80	1.60	0.000
Moderate	5.59	5.30	1.06	0.009
Bad	6.70	7.00	1.44	0.217
DIP	4.89	4.90	0.50	0.219
Good	3.43	3.10	0.78	0.001
Moderate	4.89	5.00	0.51	0.247
Bad	6.34	6.50	1.03	0.003
Comparison of total medical services provided by doctors under two payments (P)	0.009			

### 3.2 Patient benefit

The doctors’ medical decisions determined patient benefits. The maximum benefits provided to patients under both payments were 9.82. At the aggregate level, there was no statistically significant difference in the average benefits of patients between two payments. The mean benefits of patients under the FFS and DIP payments were 8.59 and 9.06, respectively. These values differed from the corresponding optimal benefits by 12.53 and 7.74%. The differences between the mean benefits and the optimal benefits under both payment systems were statistically significant.

The results indicated only patients with good health status obtained significantly fewer benefits under the FFS payment than under the DIP payment. The mean benefits for patients with the three health status under both payment systems were significantly lower than the corresponding optimal benefits. Under the FFS payment, patients with good, intermediate and poor health status experienced corresponding losses of 14.10, 10.20, and 13.23% in benefits, respectively. Under the DIP payment, patients with good, intermediate, and poor health status experienced corresponding losses of 5.70, 8.10 and 9.42% in benefits, respectively. The results indicated that as patients’ health status deteriorated, the loss in benefits of patients increased under the DIP payment. The detailed findings could be found in Table 3.

**Table 3:** Benefits obtained by patients with different health status under the FFS and DIP payments.

Variable	Mean	Median	SD	Comparison with the optimal benefits (P)
FFS	8.59	8.75	1.09	<0.001
Good	8.59	9.00	1.87	<0.001
Moderate	8.98	9.20	1.04	<0.001
Bad	8.20	8.97	1.74	<0.001
DIP	9.06	9.48	0.94	<0.001
Good	9.43	9.90	1.05	<0.001
Moderate	9.19	10.00	1.32	0.001
Bad	8.56	9.05	0.98	<0.001
Comparison of total benefits by patients under two payments (P)	0.07			

### 3.3 Physicians’ profit

Doctors’ medical decisions not only determined patient benefits, but also personal net profits. The maximum profits accrued by physicians under the FFS payment were 11.08, while under the DIP payment, the maximum profits were 11.80. At the aggregate level, the average profits obtained by physicians were 19.21% smaller under the FFS payment than under the DIP payment, and the difference was statistically significant. The physicians’ profits under the FFS and the DIP payments were 7.40 and 9.16, respectively. These values were 33.21 and 22.37% less than the maximum profits, and the differences were statistically significant.

The results indicated that physicians obtained significantly higher average profits from patients with the health status of good and moderate under the DIP payment compared to the FFS payment. But the result of patients with bad health status was opposite. Under the FFS payment, physicians’ mean profits from patients with the three health status were 45.85, 31.86 and 22.02% less than the maximum profit. Additionally, the loss of profits decreased as the physicians’ health status deteriorated. The average profits for physicians treating patients with the health status of good, moderate, and bad under the DIP payment were 10.85, 20.85, and 35.51% less than the maximum profit. As the health status of the patients deteriorated, the physicians’ loss of profit increased. The results were presented in Table 4.

**Table 4:** Physicians’ profits for patients with the different health status under the FFS and DIP payments.

Payment	Health status	Mean	Median	SD	Comparison with the optimal profits (P)
FFS	Good	6.00	5.38	2.28	<0.001
	Moderate	7.55	7.38	1.67	<0.001
	Bad	8.64	9.08	1.69	<0.001
DIP	Good	10.52	10.83	0.72	<0.001
	Moderate	9.34	9.30	0.52	<0.001
	Bad	7.61	7.55	1.28	<0.001

### 3.4 Optimal quantity decision

Overall, 43.75% of the medical decisions physicians made under the FFS payment were optimal quantity decisions, while 62.92% of the medical decisions they made under the DIP payment were optimal quantity decisions. The Pearson chi-square test indicated that the difference in the proportion of physicians who chose the optimal quantity of medical services was statistically significant under the two payment methods (p<0.001).

Patients with the three health status received different numbers of optimal health care decisions. 40.63, 47.50, 43.13% of the medical decisions physicians made for patients with good, moderate, and bad health status under the FFS payment were optimal quantity decisions, while patients with the health status of good, moderate, and bad health status received 70.00, 76.25, 42.50% of optimal quantity decisions under the DIP payment. We could know, the number of optimal quantity decisions that patients with good or moderate health status received under the DIP payment was higher than those received under the FFS payment. However, the opposite was true for patients with poor health. The difference in the proportion of optimal quantity decisions for patients with the three health conditions was statistically significant under both payment systems (*p* = 0.032).

## 4 Discussion

The results indicated that the two payment systems were associated with different provision behavior by physicians. At the general level, physicians provided significantly more services to patients than the optimal quantities under the FFS payment. This meant the FFS payment incentivizes physicians to provide more services. This finding was consistent with the conclusions found by some scholars through a controlled laboratory experiment on medical students in school (10, 11). It was the same as a field experimental study (18). Some scholars have also reached the same conclusion through empirical research and theoretical analysis (1, 19). This is because the FFS payment means that the medical insurance institution pays the expenses to the medical institution according to the actual expenses incurred by the insured person after the medical expenses are incurred. The FFS payment is a typical retrospective payment and the financial risk is mainly on the payer. Therefore, the FFS reimbursement distorts care provision by incentivizing overtreatments. Somes scholars have found that professional standards of physicians can reduce conflicts of interest between physicians and patients (20). Therefore, doctors can be regularly trained to strengthen the professional standards. The study also found that there was no statistical significance between the amounts of medical care provided by doctors and the optimal quantities under the DIP payment. It meant that physicians’ provision behavior can be regulated to a certain extent under the DIP payment. This is because the DIP payment is a typical prospective payment which forms a mechanism for the payer and the supplier to share the financial risk. During the reform from retrospective payment to prospective mode, the adjustment of incentive mechanisms for healthcare service providers’ behavior to control the cost increasing caused by the supplier-induced services. Some studies have also found that the DIP payment reform could not only effectively regulate provider behavior, but also improve the rational allocation of the regional healthcare resources (3, 5). The research results of these scholars have further verified the feasibility of the experimental economics method and the reliability of the research results.

This study indicated that patients with different health status also influenced physicians’ supply of medical services under both payment methods. Under the FFS payment, patients with the health status of good and moderate received significantly more services than the corresponding optimal care quantities. The amounts of care received by patients in bad health status were very close to the corresponding optimal quantities of care and there was no statistical relationship between them. In terms of patient benefit, the loss of benefits by patients with moderate and poor health status were smaller than the loss of benefits by patients with good health status under the FFS payment. In terms of physicians’ profit, physicians obtained higher profits when treating patients in bad health under the FFS payment than those under the DIP payment. All these indicated that the FFS payment is suitable for the treatment of patients in relatively bad health. The study (10) also found that the FFS payment is more beneficial for patients with the severe condition. The relevant studies of scholars also showed that the advantages of the FFS payment are that it can provide high-quality diagnosis and treatment services to meet the various medical service needs of patients, and also mobilize the enthusiasm of doctors (21, 22).

Under the DIP payment, patients in good health status received significantly more services than the corresponding optimal care quantities. The amounts of care received by patients with moderate health status were very close to the corresponding optimal quantities of care. Patients in bad health status gained significantly less services than the corresponding optimal quantities of care. Based on the perspective of patient benefit, with the deterioration of the disease, the benefits of the patient were decreasing. Based on the perspective of physicians’ profit, physicians obtained higher profits when treating patients with the health status of good and moderate under the DIP payment than those under the FFS payment. These results all indicated that the DIP payment is beneficial to treat patients in relatively good health status. Moreover, patients with bad health status are underserved under the DIP payment. Study by relevant scholar has also confirmed that the DIP payment is more suitable for treating patients with less severe condition (6, 15). This may be because treating mild cases does not easily exceed payment standards and hospitals need to bear losses when treating critically ill patients exceeds payment standards under the DIP payment. From a policy perspective, our results reinforce the international experience that no provider payment method is perfect (23, 24). Based on the results of this study, the following recommendations are made.

### 4.1 Optimize the multi-compound medical insurance payment and strengthen coordination in reform

Both the FFS and DIP payments have advantages. Patients in relatively bad health can be adequately treated under the FFS payment, while patients in relatively good health can get more reasonable medical services under the DIP payment. Therefore, it is necessary to continue to optimize multi-compound medical insurance payment. In this way, the advantages of the single payment method can be utilized and the balance between cost compensation and risk sharing can be achieved.

The medical insurance departments should insist on strengthening the coordination of reform and actively promote the reform of multi-compound medical insurance payment while deeply implementing the DIP payment reform. The medical insurance departments should also actively implement national medical insurance negotiation drugs policy, volume-based centralized purchasing policy and other policies to form a synergy for the reform and promote the reform of payment system to develop in depth.

### 4.2 Resolve medical insurance payment systems and performance differentially to encourage physicians to return to their jobs

This study showed that medical insurance payment systems affect physicians’ provision behavior. The most important job of doctors is healing the wounded and rescuing the dying and they should not pay too much attention to economic issues in the process of curing diseases and saving people. Therefore, it is necessary to resolve medical insurance payment systems and performance differentially, so that payment methods would not motivate doctors to make unreasonable medical services.

There are three principles healthcare providers can apply when implementing the DIP payment. First, let clinical departments pay not too much attention to the payment standards of DIP disease groups, so as to avoid affecting the treatment of patients and clinically unreasonable countermeasures. Second, through the implementation of diagnosis and treatment norms, diagnosis and treatment guidelines and so on to achieve the hospital internal homogenization management and promotion. Third, it does not directly link the overspending and balance of the DIP payment with the performance of departments and doctors.

### 4.3 Innovate the supervision of doctors’ behavior under the DIP payment

The study showed that physicians provided significantly less medical services to patients with bad health status under the DIP payment than the corresponding optimal care quantities. It meant patients with bad health status are underserved under the DIP payment. Therefore, it is necessary to innovate the supervision of doctors’ behavior under the DIP payment, such as strengthen the quality management of the first page of medical records and the settlement list of medical insurance funds. Regulatory processes should focus on changes in service capabilities, service efficiency, quality of care, cost control, patient satisfaction, and other related indicators.

## 5 Limitations

This study had the following limitations. First, we sampled only physicians from a level A of tertiary general hospital and a level A of secondary general hospital in Dongying, China. Second, the sample sizes were relatively small.

## 6 Conclusion

It found patients are overserved under the FFS payment, but patients in bad health status can receive more adequate treatment. Physicians’ provision behavior can be regulated to a certain extent under the DIP payment and the DIP payment is suitable for the treatment of patients in relatively good health status. Doctors sometimes have violations under DIP payment, such as inadequate service and so on. Therefore, it is necessary to innovate the supervision of physicians’ provision behavior under the DIP payment. It showed both medical insurance payment systems and patients with difference health status can influence physicians’ provision behavior.

## Data availability statement

The original contributions presented in the study are included in the article/Supplementary material, further inquiries can be directed to the corresponding author.

## Ethics statement

The studies involving humans were approved by the Institutional Review Board (Academic Research Ethics Committee) of Shandong University School of Public Health. The studies were conducted in accordance with the local legislation and institutional requirements. The participants provided their written informed consent to participate in this study.

## Author contributions

SL: Conceptualization, Data curation, Methodology, Writing – original draft. QS: Conceptualization, Methodology, Writing – original draft. HZ: Conceptualization, Methodology, Writing – original draft. JY: Data curation, Methodology, Writing – original draft. CZ: Conceptualization, Project administration, Supervision, Writing – review & editing.
