# Supplementary material for: How medical insurance payment systems affect the physicians’ provision behavior in China—based on experimental economics
Source: Front Public Health. 2024 May 2;12:1323090. doi: 10.3389/fpubh.2024.1323090 (PMC11097335; doi:10.3389/fpubh.2024.1323090)
Supplement: Supplementary file 1 [file Data_Sheet_1.docx]

**Supplementary Material**

**Supplementary questionnaire**

**A Study on the Influences of Medical Insurance Payment Systems on Physicians’ Behavior**

Dear physician：

The purpose of this questionnaire is to understand the impact of fee-for-service (FFS) and diagnosis-intervention package (DIP) payments on physicians’ provision behavior, so as to provide a basis for optimizing payment reform. The information in this survey is used for academic research only, not for any other purpos. There is no right or wrong answer. You just need to choose the appropriate answer for your personal situation. We sincerely appreciate your support and cooperation!

**Basic information**

1.Your Hospital ( )

2.Your gender

（1）male （2）female

3. Your age

（1） twenty to thirty years old （2） thirty-one to forty years old （3) forty-one to fifty years old （4）fifty-one to sixty years old

4. Your education

（1）college and below （2）bachelor degree （3）master's degree （4）doctoral degree

5. Your department

（1）internal medicine department （2）surgery department （3）gynecology department （4）pediatric department （5）other departments

6. Your years of employment

（1）≤five years （2）six to ten years （3）eleven to twenty years （4）＞twenty years

7. Your title

（1）junior-grade professional title （2）medium-grade professional title （3）vice senior title （4）senior title （5）other

8. Your e-mail address ( )

**Part I. Description of the experiment**

**General description**

You are participating in an economics experiment on decision making and needing to make a series of decisions on the questionnaire. If you fill out the questionnaire according to the instructions of the experiment, you can receive a certain amount of monetary income based on the decisions you make. No talking to other participants during the completion of the questionnaire. If you have any questions, please raise your hand and a panelist will come to your seat to answer them! Those who violate the rules will not be paid. All amounts in the experiment are shown in tokens. At the end of the experiment, your payment will be transferred to you at the rate of 10 tokens = 1RMB.

The experiment has two parts. The decisions you make in Part 1 have no effect on the decisions you make in Part 2，and vice versa.

**Your decisions in the first part of the experiment**

Your are doctor and no real patients existed in the experimen, you need to provide virtual patients the number of medical services. The questionnaire will show five abstract diseases A, B, C, D and E in sequential order. Each disease has 3 levels of severity: mild, moderate, and severe. Since there are 15 patient types for the combination of 5 diseases and 3 severity levels, you need to make 15 decisions in the first part. You can provide 0, 1, 2, 3, 4, 5, 6, 7, 8, 9 or 10 medical services for each different patient.

**Here is an example of the first decision scenario (fee-for-service).**


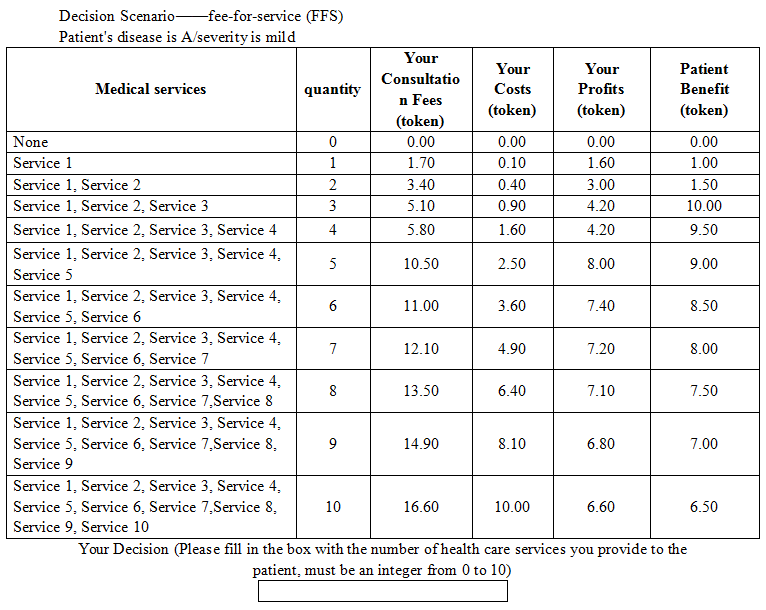


Each quantity of medical services has a corresponding consultation fee, cost, profit, and patient benefit.

Consultation fees and costs increase as the number of medical services increases. The fee minus the cost is the profit you receive.

Patient benefit refers to the extent to which patients are restored to good health by the healthcare services you provide and the cost of doing so. Higher patient benefit means better for the patient. As a result, the decisions you make about the amount of care you provide to each patient determine not only your own profit, but also the patient benefit.

You make a decision about the amount of health care to be provided to the patient by filling in an integer from 0 to 10 in the "Your Decision" column of the questionnaire. After you have made the decisions in the first part of the experiment, your payoff is the sum of the profit of the 15 decisions and is calculated at the rate of 10 tokens = 1 RMB. This remuneration is paid to you in the form of a transfer together with your remuneration for the second part of the experiment (round up to the nearest dollar).

Although there are no actual patients in the experiment, the benefits that each patient receives through the amount of care you provide will benefit a real patient. The total amount of patient benefits from your 15 decisions will be provided to a cancer patient being treated at Qilu Hospital of Shandong University, together with the total amount of patient benefits from the second part of the program, converted to RMB at the rate of 10 tokens = 1 RMB. Donation details will be sent to your email address for monitoring purposes.

**Next, please fill out and answer a few comprehension questions. They will help you familiarize yourself with the decision-making scenario.**

1. In the first part of the experiment, what is your identity? What do you need to do? Are there any real patients present in the experiment?

2. In the first part of the experiment, how many diseases does the patient have? Are they all abstract?

3. In the first part of the experiment, there are several levels of disease severity and what they are?

4. In the first part of the experiment, how are your consultation fees, your costs, and your profit calculated? How is your compensation calculated?

5. In the first part of the experiment, In the first part of the experiment, the decisions you make about the amount of care you provide to your patients determine not only your profit, but also whose benefit? Although the patients in the experiment are virtual, who in real life will benefit from the patient benefits obtained through decision making?

**Part I. Fee-for-service (FFS)**

1. Patient's disease is A/severity is mild

| **Medical services** | **quantity** | **Your Consultation Fees (token)** | **Your Costs (token)** | **Your Profits**  **(token)** | **Patient Benefit (token)** |
| --- | --- | --- | --- | --- | --- |
| None | 0 | 0.00 | 0.00 | 0.00 | 0.00 |
| Service 1 | 1 | 1.70 | 0.10 | 1.60 | 1.00 |
| Service 1, Service 2 | 2 | 3.40 | 0.40 | 3.00 | 1.50 |
| Service 1, Service 2, Service 3 | 3 | 5.10 | 0.90 | 4.20 | 10.00 |
| Service 1, Service 2, Service 3, Service 4 | 4 | 5.80 | 1.60 | 4.20 | 9.50 |
| Service 1, Service 2, Service 3, Service 4, Service 5 | 5 | 10.50 | 2.50 | 8.00 | 9.00 |
| Service 1, Service 2, Service 3, Service 4, Service 5, Service 6 | 6 | 11.00 | 3.60 | 7.40 | 8.50 |
| Service 1, Service 2, Service 3, Service 4, Service 5, Service 6, Service 7 | 7 | 12.10 | 4.90 | 7.20 | 8.00 |
| Service 1, Service 2, Service 3, Service 4, Service 5, Service 6, Service 7,Service 8 | 8 | 13.50 | 6.40 | 7.10 | 7.50 |
| Service 1, Service 2, Service 3, Service 4, Service 5, Service 6, Service 7,Service 8, Service 9 | 9 | 14.90 | 8.10 | 6.80 | 7.00 |
| Service 1, Service 2, Service 3, Service 4, Service 5, Service 6, Service 7,Service 8, Service 9, Service 10 | 10 | 16.60 | 10.00 | 6.60 | 6.50 |

Your Decision (Please fill in the box with the number of health care services you provide to the patient, must be an integer from 0 to 10)

|  |
| --- |

1. Patient's disease is B/severity is mild

| **Medical services** | **quantity** | **Your Consultation Fees (token)** | **Your Costs (token)** | **Your Profits**  **(token)** | **Patient Benefit (token)** |
| --- | --- | --- | --- | --- | --- |
| None | 0 | 0.00 | 0.00 | 0.00 | 0.00 |
| Service 1 | 1 | 1.00 | 0.10 | 0.90 | 1.00 |
| Service 1, Service 2 | 2 | 2.40 | 0.40 | 2.00 | 1.50 |
| Service 1, Service 2, Service 3 | 3 | 3.50 | 0.90 | 2.60 | 10.00 |
| Service 1, Service 2, Service 3, Service 4 | 4 | 8.00 | 1.60 | 6.40 | 9.50 |
| Service 1, Service 2, Service 3, Service 4, Service 5 | 5 | 8.40 | 2.50 | 5.90 | 9.00 |
| Service 1, Service 2, Service 3, Service 4, Service 5, Service 6 | 6 | 9.40 | 3.60 | 5.80 | 8.50 |
| Service 1, Service 2, Service 3, Service 4, Service 5, Service 6, Service 7 | 7 | 16.00 | 4.90 | 11.10 | 8.00 |
| Service 1, Service 2, Service 3, Service 4, Service 5, Service 6, Service 7,Service 8 | 8 | 18.00 | 6.40 | 11.60 | 7.50 |
| Service 1, Service 2, Service 3, Service 4, Service 5, Service 6, Service 7,Service 8, Service 9 | 9 | 20.00 | 8.10 | 11.90 | 7.00 |
| Service 1, Service 2, Service 3, Service 4, Service 5, Service 6, Service 7,Service 8, Service 9, Service 10 | 10 | 22.50 | 10.00 | 12.50 | 6.50 |

Your Decision (Please fill in the box with the number of health care services you provide to the patient, must be an integer from 0 to 10)

|  |
| --- |

1. Patient's disease is C/severity is mild

| **Medical services** | **quantity** | **Your Consultation Fees (token)** | **Your Costs (token)** | **Your Profits**  **(token)** | **Patient Benefit (token)** |
| --- | --- | --- | --- | --- | --- |
| None | 0 | 0.00 | 0.00 | 0.00 | 0.00 |
| Service 1 | 1 | 1.80 | 0.10 | 1.70 | 1.00 |
| Service 1, Service 2 | 2 | 3.60 | 0.40 | 3.20 | 1.50 |
| Service 1, Service 2, Service 3 | 3 | 5.40 | 0.90 | 4.50 | 10.00 |
| Service 1, Service 2, Service 3, Service 4 | 4 | 7.20 | 1.60 | 5.60 | 9.50 |
| Service 1, Service 2, Service 3, Service 4, Service 5 | 5 | 9.00 | 2.50 | 6.50 | 9.00 |
| Service 1, Service 2, Service 3, Service 4, Service 5, Service 6 | 6 | 10.80 | 3.60 | 7.20 | 8.50 |
| Service 1, Service 2, Service 3, Service 4, Service 5, Service 6, Service 7 | 7 | 12.60 | 4.90 | 7.70 | 8.00 |
| Service 1, Service 2, Service 3, Service 4, Service 5, Service 6, Service 7,Service 8 | 8 | 14.40 | 6.40 | 8.00 | 7.50 |
| Service 1, Service 2, Service 3, Service 4, Service 5, Service 6, Service 7,Service 8, Service 9 | 9 | 16.20 | 8.10 | 8.10 | 7.00 |
| Service 1, Service 2, Service 3, Service 4, Service 5, Service 6, Service 7,Service 8, Service 9, Service 10 | 10 | 18.30 | 10.00 | 8.30 | 6.50 |

Your Decision (Please fill in the box with the number of health care services you provide to the patient, must be an integer from 0 to 10)

|  |
| --- |

1. Patient's disease is D/severity is mild

| **Medical services** | **quantity** | **Your Consultation Fees (token)** | **Your Costs (token)** | **Your Profits**  **(token)** | **Patient Benefit (token)** |
| --- | --- | --- | --- | --- | --- |
| None | 0 | 0.00 | 0.00 | 0.00 | 0.00 |
| Service 1 | 1 | 2.00 | 0.10 | 1.90 | 1.00 |
| Service 1, Service 2 | 2 | 4.00 | 0.40 | 3.60 | 1.50 |
| Service 1, Service 2, Service 3 | 3 | 6.00 | 0.90 | 5.10 | 10.00 |
| Service 1, Service 2, Service 3, Service 4 | 4 | 8.00 | 1.60 | 6.40 | 9.50 |
| Service 1, Service 2, Service 3, Service 4, Service 5 | 5 | 8.20 | 2.50 | 5.70 | 9.00 |
| Service 1, Service 2, Service 3, Service 4, Service 5, Service 6 | 6 | 15.00 | 3.60 | 11.40 | 8.50 |
| Service 1, Service 2, Service 3, Service 4, Service 5, Service 6, Service 7 | 7 | 16.90 | 4.90 | 12.00 | 8.00 |
| Service 1, Service 2, Service 3, Service 4, Service 5, Service 6, Service 7,Service 8 | 8 | 18.90 | 6.40 | 12.50 | 7.50 |
| Service 1, Service 2, Service 3, Service 4, Service 5, Service 6, Service 7,Service 8, Service 9 | 9 | 21.30 | 8.10 | 13.20 | 7.00 |
| Service 1, Service 2, Service 3, Service 4, Service 5, Service 6, Service 7,Service 8, Service 9, Service 10 | 10 | 23.60 | 10.00 | 13.60 | 6.50 |

Your Decision (Please fill in the box with the number of health care services you provide to the patient, must be an integer from 0 to 10)

|  |
| --- |

1. Patient's disease is E/severity is mild

| **Medical services** | **quantity** | **Your Consultation Fees (token)** | **Your Costs (token)** | **Your Profits**  **(token)** | **Patient Benefit (token)** |
| --- | --- | --- | --- | --- | --- |
| None | 0 | 0.00 | 0.00 | 0.00 | 0.00 |
| Service 1 | 1 | 1.00 | 0.10 | 0.90 | 1.00 |
| Service 1, Service 2 | 2 | 2.00 | 0.40 | 1.60 | 1.50 |
| Service 1, Service 2, Service 3 | 3 | 6.00 | 0.90 | 5.10 | 10.00 |
| Service 1, Service 2, Service 3, Service 4 | 4 | 6.70 | 1.60 | 5.10 | 9.50 |
| Service 1, Service 2, Service 3, Service 4, Service 5 | 5 | 7.60 | 2.50 | 5.10 | 9.00 |
| Service 1, Service 2, Service 3, Service 4, Service 5, Service 6 | 6 | 11.00 | 3.60 | 7.40 | 8.50 |
| Service 1, Service 2, Service 3, Service 4, Service 5, Service 6, Service 7 | 7 | 12.30 | 4.90 | 7.40 | 8.00 |
| Service 1, Service 2, Service 3, Service 4, Service 5, Service 6, Service 7,Service 8 | 8 | 18.00 | 6.40 | 11.60 | 7.50 |
| Service 1, Service 2, Service 3, Service 4, Service 5, Service 6, Service 7,Service 8, Service 9 | 9 | 20.50 | 8.10 | 12.40 | 7.00 |
| Service 1, Service 2, Service 3, Service 4, Service 5, Service 6, Service 7,Service 8, Service 9, Service 10 | 10 | 23.00 | 10.00 | 13.00 | 6.50 |

Your Decision (Please fill in the box with the number of health care services you provide to the patient, must be an integer from 0 to 10)

|  |
| --- |

1. Patient's disease is A/severity is moderate

| **Medical services** | **quantity** | **Your Consultation Fees (token)** | **Your Costs (token)** | **Your Profits**  **(token)** | **Patient Benefit (token)** |
| --- | --- | --- | --- | --- | --- |
| None | 0 | 0.00 | 0.00 | 0.00 | 0.00 |
| Service 1 | 1 | 1.70 | 0.10 | 1.60 | 0.75 |
| Service 1, Service 2 | 2 | 3.40 | 0.40 | 3.00 | 1.50 |
| Service 1, Service 2, Service 3 | 3 | 5.10 | 0.90 | 4.20 | 2.00 |
| Service 1, Service 2, Service 3, Service 4 | 4 | 5.80 | 1.60 | 4.20 | 7.00 |
| Service 1, Service 2, Service 3, Service 4, Service 5 | 5 | 10.50 | 2.50 | 8.00 | 10.00 |
| Service 1, Service 2, Service 3, Service 4, Service 5, Service 6 | 6 | 11.00 | 3.60 | 7.40 | 9.50 |
| Service 1, Service 2, Service 3, Service 4, Service 5, Service 6, Service 7 | 7 | 12.10 | 4.90 | 7.20 | 9.00 |
| Service 1, Service 2, Service 3, Service 4, Service 5, Service 6, Service 7,Service 8 | 8 | 13.50 | 6.40 | 7.10 | 8.50 |
| Service 1, Service 2, Service 3, Service 4, Service 5, Service 6, Service 7,Service 8, Service 9 | 9 | 14.90 | 8.10 | 6.80 | 8.00 |
| Service 1, Service 2, Service 3, Service 4, Service 5, Service 6, Service 7,Service 8, Service 9, Service 10 | 10 | 16.60 | 10.00 | 6.60 | 7.50 |

Your Decision (Please fill in the box with the number of health care services you provide to the patient, must be an integer from 0 to 10)

|  |
| --- |

1. Patient's disease is B/severity is moderate

| **Medical services** | **quantity** | **Your Consultation Fees (token)** | **Your Costs (token)** | **Your Profits**  **(token)** | **Patient Benefit (token)** |
| --- | --- | --- | --- | --- | --- |
| None | 0 | 0.00 | 0.00 | 0.00 | 0.00 |
| Service 1 | 1 | 1.00 | 0.10 | 0.90 | 0.75 |
| Service 1, Service 2 | 2 | 2.40 | 0.40 | 2.00 | 1.50 |
| Service 1, Service 2, Service 3 | 3 | 3.50 | 0.90 | 2.60 | 2.00 |
| Service 1, Service 2, Service 3, Service 4 | 4 | 8.00 | 1.60 | 6.40 | 7.00 |
| Service 1, Service 2, Service 3, Service 4, Service 5 | 5 | 8.40 | 2.50 | 5.90 | 10.00 |
| Service 1, Service 2, Service 3, Service 4, Service 5, Service 6 | 6 | 9.40 | 3.60 | 5.80 | 9.50 |
| Service 1, Service 2, Service 3, Service 4, Service 5, Service 6, Service 7 | 7 | 16.00 | 4.90 | 11.10 | 9.00 |
| Service 1, Service 2, Service 3, Service 4, Service 5, Service 6, Service 7,Service 8 | 8 | 18.00 | 6.40 | 11.60 | 8.50 |
| Service 1, Service 2, Service 3, Service 4, Service 5, Service 6, Service 7,Service 8, Service 9 | 9 | 20.00 | 8.10 | 11.90 | 8.00 |
| Service 1, Service 2, Service 3, Service 4, Service 5, Service 6, Service 7,Service 8, Service 9, Service 10 | 10 | 22.50 | 10.00 | 12.50 | 7.50 |

Your Decision (Please fill in the box with the number of health care services you provide to the patient, must be an integer from 0 to 10)

|  |
| --- |

1. Patient's disease is C/severity is moderate

| **Medical services** | **quantity** | **Your Consultation Fees (token)** | **Your Costs (token)** | **Your Profits**  **(token)** | **Patient Benefit (token)** |
| --- | --- | --- | --- | --- | --- |
| None | 0 | 0.00 | 0.00 | 0.00 | 0.00 |
| Service 1 | 1 | 1.80 | 0.10 | 1.70 | 0.75 |
| Service 1, Service 2 | 2 | 3.60 | 0.40 | 3.20 | 1.50 |
| Service 1, Service 2, Service 3 | 3 | 5.40 | 0.90 | 4.50 | 2.00 |
| Service 1, Service 2, Service 3, Service 4 | 4 | 7.20 | 1.60 | 5.60 | 7.00 |
| Service 1, Service 2, Service 3, Service 4, Service 5 | 5 | 9.00 | 2.50 | 6.50 | 10.00 |
| Service 1, Service 2, Service 3, Service 4, Service 5, Service 6 | 6 | 10.80 | 3.60 | 7.20 | 9.50 |
| Service 1, Service 2, Service 3, Service 4, Service 5, Service 6, Service 7 | 7 | 12.60 | 4.90 | 7.70 | 9.00 |
| Service 1, Service 2, Service 3, Service 4, Service 5, Service 6, Service 7,Service 8 | 8 | 14.40 | 6.40 | 8.00 | 8.50 |
| Service 1, Service 2, Service 3, Service 4, Service 5, Service 6, Service 7,Service 8, Service 9 | 9 | 16.20 | 8.10 | 8.10 | 8.00 |
| Service 1, Service 2, Service 3, Service 4, Service 5, Service 6, Service 7,Service 8, Service 9, Service 10 | 10 | 18.30 | 10.00 | 8.30 | 7.50 |

Your Decision (Please fill in the box with the number of health care services you provide to the patient, must be an integer from 0 to 10)

|  |
| --- |

1. Patient's disease is D/severity is moderate

| **Medical services** | **quantity** | **Your Consultation Fees (token)** | **Your Costs (token)** | **Your Profits**  **(token)** | **Patient Benefit (token)** |
| --- | --- | --- | --- | --- | --- |
| None | 0 | 0.00 | 0.00 | 0.00 | 0.00 |
| Service 1 | 1 | 2.00 | 0.10 | 1.90 | 0.75 |
| Service 1, Service 2 | 2 | 4.00 | 0.40 | 3.60 | 1.50 |
| Service 1, Service 2, Service 3 | 3 | 6.00 | 0.90 | 5.10 | 2.00 |
| Service 1, Service 2, Service 3, Service 4 | 4 | 8.00 | 1.60 | 6.40 | 7.00 |
| Service 1, Service 2, Service 3, Service 4, Service 5 | 5 | 8.20 | 2.50 | 5.70 | 10.00 |
| Service 1, Service 2, Service 3, Service 4, Service 5, Service 6 | 6 | 15.00 | 3.60 | 11.40 | 9.50 |
| Service 1, Service 2, Service 3, Service 4, Service 5, Service 6, Service 7 | 7 | 16.90 | 4.90 | 12.00 | 9.00 |
| Service 1, Service 2, Service 3, Service 4, Service 5, Service 6, Service 7,Service 8 | 8 | 18.90 | 6.40 | 12.50 | 8.50 |
| Service 1, Service 2, Service 3, Service 4, Service 5, Service 6, Service 7,Service 8, Service 9 | 9 | 21.30 | 8.10 | 13.20 | 8.00 |
| Service 1, Service 2, Service 3, Service 4, Service 5, Service 6, Service 7,Service 8, Service 9, Service 10 | 10 | 23.60 | 10.00 | 13.60 | 7.50 |

Your Decision (Please fill in the box with the number of health care services you provide to the patient, must be an integer from 0 to 10)

|  |
| --- |

1. Patient's disease is E/severity is moderate

| **Medical services** | **quantity** | **Your Consultation Fees (token)** | **Your Costs (token)** | **Your Profits**  **(token)** | **Patient Benefit (token)** |
| --- | --- | --- | --- | --- | --- |
| None | 0 | 0.00 | 0.00 | 0.00 | 0.00 |
| Service 1 | 1 | 1.00 | 0.10 | 0.90 | 0.75 |
| Service 1, Service 2 | 2 | 2.00 | 0.40 | 1.60 | 1.50 |
| Service 1, Service 2, Service 3 | 3 | 6.00 | 0.90 | 5.10 | 2.00 |
| Service 1, Service 2, Service 3, Service 4 | 4 | 6.70 | 1.60 | 5.10 | 7.00 |
| Service 1, Service 2, Service 3, Service 4, Service 5 | 5 | 7.60 | 2.50 | 5.10 | 10.00 |
| Service 1, Service 2, Service 3, Service 4, Service 5, Service 6 | 6 | 11.00 | 3.60 | 7.40 | 9.50 |
| Service 1, Service 2, Service 3, Service 4, Service 5, Service 6, Service 7 | 7 | 12.30 | 4.90 | 7.40 | 9.00 |
| Service 1, Service 2, Service 3, Service 4, Service 5, Service 6, Service 7,Service 8 | 8 | 18.00 | 6.40 | 11.60 | 8.50 |
| Service 1, Service 2, Service 3, Service 4, Service 5, Service 6, Service 7,Service 8, Service 9 | 9 | 20.50 | 8.10 | 12.40 | 8.00 |
| Service 1, Service 2, Service 3, Service 4, Service 5, Service 6, Service 7,Service 8, Service 9, Service 10 | 10 | 23.00 | 10.00 | 13.00 | 7.50 |

Your Decision (Please fill in the box with the number of health care services you provide to the patient, must be an integer from 0 to 10)

|  |
| --- |

1. Patient's disease is A/severity is severe

| **Medical services** | **quantity** | **Your Consultation Fees (token)** | **Your Costs (token)** | **Your Profits**  **(token)** | **Patient Benefit (token)** |
| --- | --- | --- | --- | --- | --- |
| None | 0 | 0.00 | 0.00 | 0.00 | 0.00 |
| Service 1 | 1 | 1.70 | 0.10 | 1.60 | 0.75 |
| Service 1, Service 2 | 2 | 3.40 | 0.40 | 3.00 | 2.20 |
| Service 1, Service 2, Service 3 | 3 | 5.10 | 0.90 | 4.20 | 4.05 |
| Service 1, Service 2, Service 3, Service 4 | 4 | 5.80 | 1.60 | 4.20 | 6.00 |
| Service 1, Service 2, Service 3, Service 4, Service 5 | 5 | 10.50 | 2.50 | 8.00 | 7.75 |
| Service 1, Service 2, Service 3, Service 4, Service 5, Service 6 | 6 | 11.00 | 3.60 | 7.40 | 9.00 |
| Service 1, Service 2, Service 3, Service 4, Service 5, Service 6, Service 7 | 7 | 12.10 | 4.90 | 7.20 | 9.45 |
| Service 1, Service 2, Service 3, Service 4, Service 5, Service 6, Service 7,Service 8 | 8 | 13.50 | 6.40 | 7.10 | 8.80 |
| Service 1, Service 2, Service 3, Service 4, Service 5, Service 6, Service 7,Service 8, Service 9 | 9 | 14.90 | 8.10 | 6.80 | 6.75 |
| Service 1, Service 2, Service 3, Service 4, Service 5, Service 6, Service 7,Service 8, Service 9, Service 10 | 10 | 16.60 | 10.00 | 6.60 | 3.00 |

Your Decision (Please fill in the box with the number of health care services you provide to the patient, must be an integer from 0 to 10)

|  |
| --- |

1. Patient's disease is B/severity is severe

| **Medical services** | **quantity** | **Your Consultation Fees (token)** | **Your Costs (token)** | **Your Profits**  **(token)** | **Patient Benefit (token)** |
| --- | --- | --- | --- | --- | --- |
| None | 0 | 0.00 | 0.00 | 0.00 | 0.00 |
| Service 1 | 1 | 1.00 | 0.10 | 0.90 | 0.75 |
| Service 1, Service 2 | 2 | 2.40 | 0.40 | 2.00 | 2.20 |
| Service 1, Service 2, Service 3 | 3 | 3.50 | 0.90 | 2.60 | 4.05 |
| Service 1, Service 2, Service 3, Service 4 | 4 | 8.00 | 1.60 | 6.40 | 6.00 |
| Service 1, Service 2, Service 3, Service 4, Service 5 | 5 | 8.40 | 2.50 | 5.90 | 7.75 |
| Service 1, Service 2, Service 3, Service 4, Service 5, Service 6 | 6 | 9.40 | 3.60 | 5.80 | 9.00 |
| Service 1, Service 2, Service 3, Service 4, Service 5, Service 6, Service 7 | 7 | 16.00 | 4.90 | 11.10 | 9.45 |
| Service 1, Service 2, Service 3, Service 4, Service 5, Service 6, Service 7,Service 8 | 8 | 18.00 | 6.40 | 11.60 | 8.80 |
| Service 1, Service 2, Service 3, Service 4, Service 5, Service 6, Service 7,Service 8, Service 9 | 9 | 20.00 | 8.10 | 11.90 | 6.75 |
| Service 1, Service 2, Service 3, Service 4, Service 5, Service 6, Service 7,Service 8, Service 9, Service 10 | 10 | 22.50 | 10.00 | 12.50 | 3.00 |

Your Decision (Please fill in the box with the number of health care services you provide to the patient, must be an integer from 0 to 10)

|  |
| --- |

1. Patient's disease is C/severity is severe

| **Medical services** | **quantity** | **Your Consultation Fees (token)** | **Your Costs (token)** | **Your Profits**  **(token)** | **Patient Benefit (token)** |
| --- | --- | --- | --- | --- | --- |
| None | 0 | 0.00 | 0.00 | 0.00 | 0.00 |
| Service 1 | 1 | 1.80 | 0.10 | 1.70 | 0.75 |
| Service 1, Service 2 | 2 | 3.60 | 0.40 | 3.20 | 2.20 |
| Service 1, Service 2, Service 3 | 3 | 5.40 | 0.90 | 4.50 | 4.05 |
| Service 1, Service 2, Service 3, Service 4 | 4 | 7.20 | 1.60 | 5.60 | 6.00 |
| Service 1, Service 2, Service 3, Service 4, Service 5 | 5 | 9.00 | 2.50 | 6.50 | 7.75 |
| Service 1, Service 2, Service 3, Service 4, Service 5, Service 6 | 6 | 10.80 | 3.60 | 7.20 | 9.00 |
| Service 1, Service 2, Service 3, Service 4, Service 5, Service 6, Service 7 | 7 | 12.60 | 4.90 | 7.70 | 9.45 |
| Service 1, Service 2, Service 3, Service 4, Service 5, Service 6, Service 7,Service 8 | 8 | 14.40 | 6.40 | 8.00 | 8.80 |
| Service 1, Service 2, Service 3, Service 4, Service 5, Service 6, Service 7,Service 8, Service 9 | 9 | 16.20 | 8.10 | 8.10 | 6.75 |
| Service 1, Service 2, Service 3, Service 4, Service 5, Service 6, Service 7,Service 8, Service 9, Service 10 | 10 | 18.30 | 10.00 | 8.30 | 3.00 |

Your Decision (Please fill in the box with the number of health care services you provide to the patient, must be an integer from 0 to 10)

|  |
| --- |

1. Patient's disease is D/severity is severe

| **Medical services** | **quantity** | **Your Consultation Fees (token)** | **Your Costs (token)** | **Your Profits**  **(token)** | **Patient Benefit (token)** |
| --- | --- | --- | --- | --- | --- |
| None | 0 | 0.00 | 0.00 | 0.00 | 0.00 |
| Service 1 | 1 | 2.00 | 0.10 | 1.90 | 0.75 |
| Service 1, Service 2 | 2 | 4.00 | 0.40 | 3.60 | 2.20 |
| Service 1, Service 2, Service 3 | 3 | 6.00 | 0.90 | 5.10 | 4.05 |
| Service 1, Service 2, Service 3, Service 4 | 4 | 8.00 | 1.60 | 6.40 | 6.00 |
| Service 1, Service 2, Service 3, Service 4, Service 5 | 5 | 8.20 | 2.50 | 5.70 | 7.75 |
| Service 1, Service 2, Service 3, Service 4, Service 5, Service 6 | 6 | 15.00 | 3.60 | 11.40 | 9.00 |
| Service 1, Service 2, Service 3, Service 4, Service 5, Service 6, Service 7 | 7 | 16.90 | 4.90 | 12.00 | 9.45 |
| Service 1, Service 2, Service 3, Service 4, Service 5, Service 6, Service 7,Service 8 | 8 | 18.90 | 6.40 | 12.50 | 8.80 |
| Service 1, Service 2, Service 3, Service 4, Service 5, Service 6, Service 7,Service 8, Service 9 | 9 | 21.30 | 8.10 | 13.20 | 6.75 |
| Service 1, Service 2, Service 3, Service 4, Service 5, Service 6, Service 7,Service 8, Service 9, Service 10 | 10 | 23.60 | 10.00 | 13.60 | 3.00 |

Your Decision (Please fill in the box with the number of health care services you provide to the patient, must be an integer from 0 to 10)

|  |
| --- |

1. Patient's disease is E/severity is severe

| **Medical services** | **quantity** | **Your Consultation Fees (token)** | **Your Costs (token)** | **Your Profits**  **(token)** | **Patient Benefit (token)** |
| --- | --- | --- | --- | --- | --- |
| None | 0 | 0.00 | 0.00 | 0.00 | 0.00 |
| Service 1 | 1 | 1.00 | 0.10 | 0.90 | 0.75 |
| Service 1, Service 2 | 2 | 2.00 | 0.40 | 1.60 | 2.20 |
| Service 1, Service 2, Service 3 | 3 | 6.00 | 0.90 | 5.10 | 4.05 |
| Service 1, Service 2, Service 3, Service 4 | 4 | 6.70 | 1.60 | 5.10 | 6.00 |
| Service 1, Service 2, Service 3, Service 4, Service 5 | 5 | 7.60 | 2.50 | 5.10 | 7.75 |
| Service 1, Service 2, Service 3, Service 4, Service 5, Service 6 | 6 | 11.00 | 3.60 | 7.40 | 9.00 |
| Service 1, Service 2, Service 3, Service 4, Service 5, Service 6, Service 7 | 7 | 12.30 | 4.90 | 7.40 | 9.45 |
| Service 1, Service 2, Service 3, Service 4, Service 5, Service 6, Service 7,Service 8 | 8 | 18.00 | 6.40 | 11.60 | 8.80 |
| Service 1, Service 2, Service 3, Service 4, Service 5, Service 6, Service 7,Service 8, Service 9 | 9 | 20.50 | 8.10 | 12.40 | 6.75 |
| Service 1, Service 2, Service 3, Service 4, Service 5, Service 6, Service 7,Service 8, Service 9, Service 10 | 10 | 23.00 | 10.00 | 13.00 | 3.00 |

Your Decision (Please fill in the box with the number of health care services you provide to the patient, must be an integer from 0 to 10)

|  |
| --- |

**Part II. Description of the experiment**

In the second part of the experiment, you still have to make 15 decisions, and the general instructions from the first part of the experiment are still valid for the second part.

**Your decisions in the second part of the experiment**

Your identity remains a physician and no real patients present in the second part of the experiment, you need to provide virtual patients the number of medical services. The questionnaire will show five abstract diseases A, B, C, D and E in sequential order. You can provide 0, 1, 2, 3, 4, 5, 6, 7, 8, 9 or 10 medical services for each different patient.

**Here is an example of the second decision scenario (diagnosis-intervention package).**


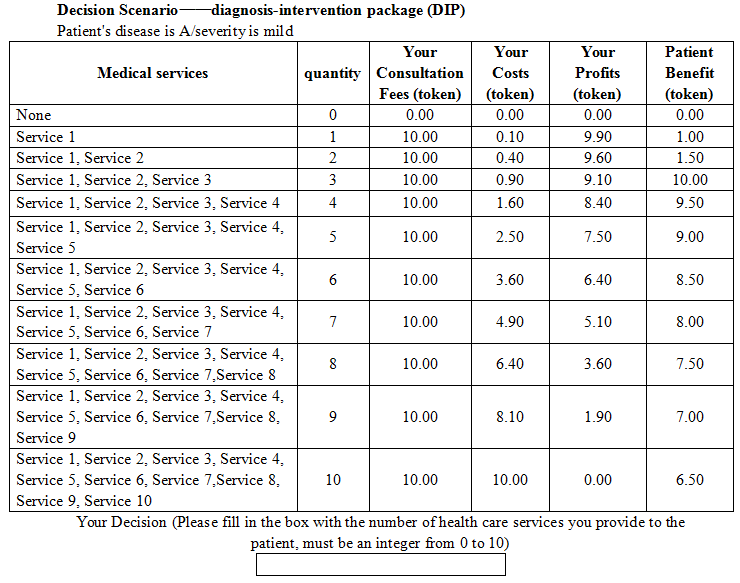


Each quantity of medical services has a corresponding consultation fee, cost, profit, and patient benefit.

You will receive a fixed amount for each patient treated for the same disease, regardless of the number of medical services.

Your costs increase as the number of medical services increases. The fee minus the cost is the profit you receive.

Patient benefit refers to the extent to which patients are restored to good health by the healthcare services you provide and the cost of doing so. Higher patient benefit means better for the patient. As a result, the decisions you make about the amount of care you provide to each patient determine not only your own profit, but also the patient benefit.

You make a decision about the amount of health care to be provided to the patient by filling in an integer from 0 to 10 in the "Your Decision" column of the questionnaire. After you have made the decisions in the second part of the experiment, your payoff is the sum of the profit of the 15 decisions and is calculated at the rate of 10 tokens = 1 RMB. This remuneration is paid to you in the form of a transfer together with your remuneration for the first part of the experiment (round up to the nearest dollar).

Although there are no actual patients in the experiment, the benefits that each patient receives through the amount of care you provide will benefit a real patient. The total amount of patient benefits from your 15 decisions will be provided to a cancer patient being treated at Qilu Hospital of Shandong University, together with the total amount of patient benefits from the first part of the program, converted to RMB at the rate of 10 tokens = 1 RMB. Donation details will be sent to your email address for monitoring purposes.

**Next, please fill out and answer a few comprehension questions. They will help you familiarize yourself with the decision-making scenario.**

1. In the second part of the experiment, what is your identity? What do you need to do? Are there any real patients present in the experiment?

2. In the second part of the experiment, how many diseases does the patient have? Are they all abstract?

3. In the second part of the experiment, there are several levels of disease severity and what they are?

4. In the second part of the experiment, how are your consultation fees, your costs, and your profit calculated? How is your compensation calculated?

5. In the second part of the experiment, In the first part of the experiment, the decisions you make about the amount of care you provide to your patients determine not only your profit, but also whose benefit? Although the patients in the experiment are virtual, who in real life will benefit from the patient benefits obtained through decision making?

**Part II. Diagnosis-intervention package (DIP)**

1.Patient's disease is A/severity is mild

| **Medical services** | **quantity** | **Your Consultation Fees (token)** | **Your Costs (token)** | **Your Profits**  **(token)** | **Patient Benefit (token)** |
| --- | --- | --- | --- | --- | --- |
| None | 0 | 0.00 | 0.00 | 0.00 | 0.00 |
| Service 1 | 1 | 10.00 | 0.10 | 9.90 | 1.00 |
| Service 1, Service 2 | 2 | 10.00 | 0.40 | 9.60 | 1.50 |
| Service 1, Service 2, Service 3 | 3 | 10.00 | 0.90 | 9.10 | 10.00 |
| Service 1, Service 2, Service 3, Service 4 | 4 | 10.00 | 1.60 | 8.40 | 9.50 |
| Service 1, Service 2, Service 3, Service 4, Service 5 | 5 | 10.00 | 2.50 | 7.50 | 9.00 |
| Service 1, Service 2, Service 3, Service 4, Service 5, Service 6 | 6 | 10.00 | 3.60 | 6.40 | 8.50 |
| Service 1, Service 2, Service 3, Service 4, Service 5, Service 6, Service 7 | 7 | 10.00 | 4.90 | 5.10 | 8.00 |
| Service 1, Service 2, Service 3, Service 4, Service 5, Service 6, Service 7,Service 8 | 8 | 10.00 | 6.40 | 3.60 | 7.50 |
| Service 1, Service 2, Service 3, Service 4, Service 5, Service 6, Service 7,Service 8, Service 9 | 9 | 10.00 | 8.10 | 1.90 | 7.00 |
| Service 1, Service 2, Service 3, Service 4, Service 5, Service 6, Service 7,Service 8, Service 9, Service 10 | 10 | 10.00 | 10.00 | 0.00 | 6.50 |

Your Decision (Please fill in the box with the number of health care services you provide to the patient, must be an integer from 0 to 10)

|  |
| --- |

2.Patient's disease is B/severity is mild

| **Medical services** | **quantity** | **Your Consultation Fees (token)** | **Your Costs (token)** | **Your Profits**  **(token)** | **Patient Benefit (token)** |
| --- | --- | --- | --- | --- | --- |
| None | 0 | 0.00 | 0.00 | 0.00 | 0.00 |
| Service 1 | 1 | 13.00 | 0.10 | 12.90 | 1.00 |
| Service 1, Service 2 | 2 | 13.00 | 0.40 | 12.60 | 1.50 |
| Service 1, Service 2, Service 3 | 3 | 13.00 | 0.90 | 12.10 | 10.00 |
| Service 1, Service 2, Service 3, Service 4 | 4 | 13.00 | 1.60 | 11.40 | 9.50 |
| Service 1, Service 2, Service 3, Service 4, Service 5 | 5 | 13.00 | 2.50 | 10.50 | 9.00 |
| Service 1, Service 2, Service 3, Service 4, Service 5, Service 6 | 6 | 13.00 | 3.60 | 9.40 | 8.50 |
| Service 1, Service 2, Service 3, Service 4, Service 5, Service 6, Service 7 | 7 | 13.00 | 4.90 | 8.10 | 8.00 |
| Service 1, Service 2, Service 3, Service 4, Service 5, Service 6, Service 7,Service 8 | 8 | 13.00 | 6.40 | 6.60 | 7.50 |
| Service 1, Service 2, Service 3, Service 4, Service 5, Service 6, Service 7,Service 8, Service 9 | 9 | 13.00 | 8.10 | 4.90 | 7.00 |
| Service 1, Service 2, Service 3, Service 4, Service 5, Service 6, Service 7,Service 8, Service 9, Service 10 | 10 | 13.00 | 10.00 | 3.00 | 6.50 |

Your Decision (Please fill in the box with the number of health care services you provide to the patient, must be an integer from 0 to 10)

|  |
| --- |

3.Patient's disease is C/severity is mild

| **Medical services** | **quantity** | **Your Consultation Fees (token)** | **Your Costs (token)** | **Your Profits**  **(token)** | **Patient Benefit (token)** |
| --- | --- | --- | --- | --- | --- |
| None | 0 | 0.00 | 0.00 | 0.00 | 0.00 |
| Service 1 | 1 | 9.00 | 0.10 | 8.90 | 1.00 |
| Service 1, Service 2 | 2 | 9.00 | 0.40 | 8.60 | 1.50 |
| Service 1, Service 2, Service 3 | 3 | 9.00 | 0.90 | 8.10 | 10.00 |
| Service 1, Service 2, Service 3, Service 4 | 4 | 9.00 | 1.60 | 7.40 | 9.50 |
| Service 1, Service 2, Service 3, Service 4, Service 5 | 5 | 9.00 | 2.50 | 6.50 | 9.00 |
| Service 1, Service 2, Service 3, Service 4, Service 5, Service 6 | 6 | 9.00 | 3.60 | 5.40 | 8.50 |
| Service 1, Service 2, Service 3, Service 4, Service 5, Service 6, Service 7 | 7 | 9.00 | 4.90 | 4.10 | 8.00 |
| Service 1, Service 2, Service 3, Service 4, Service 5, Service 6, Service 7,Service 8 | 8 | 9.00 | 6.40 | 2.60 | 7.50 |
| Service 1, Service 2, Service 3, Service 4, Service 5, Service 6, Service 7,Service 8, Service 9 | 9 | 9.00 | 8.10 | 0.90 | 7.00 |
| Service 1, Service 2, Service 3, Service 4, Service 5, Service 6, Service 7,Service 8, Service 9, Service 10 | 10 | 9.00 | 10.00 | -1.00 | 6.50 |

Your Decision (Please fill in the box with the number of health care services you provide to the patient, must be an integer from 0 to 10)

|  |
| --- |

1. Patient's disease is D/severity is mild

| **Medical services** | **quantity** | **Your Consultation Fees (token)** | **Your Costs (token)** | **Your Profits**  **(token)** | **Patient Benefit (token)** |
| --- | --- | --- | --- | --- | --- |
| None | 0 | 0.00 | 0.00 | 0.00 | 0.00 |
| Service 1 | 1 | 15.00 | 0.10 | 14.90 | 1.00 |
| Service 1, Service 2 | 2 | 15.00 | 0.40 | 14.60 | 1.50 |
| Service 1, Service 2, Service 3 | 3 | 15.00 | 0.90 | 14.10 | 10.00 |
| Service 1, Service 2, Service 3, Service 4 | 4 | 15.00 | 1.60 | 13.40 | 9.50 |
| Service 1, Service 2, Service 3, Service 4, Service 5 | 5 | 15.00 | 2.50 | 12.50 | 9.00 |
| Service 1, Service 2, Service 3, Service 4, Service 5, Service 6 | 6 | 15.00 | 3.60 | 11.40 | 8.50 |
| Service 1, Service 2, Service 3, Service 4, Service 5, Service 6, Service 7 | 7 | 15.00 | 4.90 | 10.10 | 8.00 |
| Service 1, Service 2, Service 3, Service 4, Service 5, Service 6, Service 7,Service 8 | 8 | 15.00 | 6.40 | 8.60 | 7.50 |
| Service 1, Service 2, Service 3, Service 4, Service 5, Service 6, Service 7,Service 8, Service 9 | 9 | 15.00 | 8.10 | 6.90 | 7.00 |
| Service 1, Service 2, Service 3, Service 4, Service 5, Service 6, Service 7,Service 8, Service 9, Service 10 | 10 | 15.00 | 10.00 | 5.00 | 6.50 |

Your Decision (Please fill in the box with the number of health care services you provide to the patient, must be an integer from 0 to 10)

|  |
| --- |

1. Patient's disease is E/severity is mild

| **Medical services** | **quantity** | **Your Consultation Fees (token)** | **Your Costs (token)** | **Your Profits**  **(token)** | **Patient Benefit (token)** |
| --- | --- | --- | --- | --- | --- |
| None | 0 | 0.00 | 0.00 | 0.00 | 0.00 |
| Service 1 | 1 | 12.00 | 0.10 | 11.90 | 1.00 |
| Service 1, Service 2 | 2 | 12.00 | 0.40 | 11.60 | 1.50 |
| Service 1, Service 2, Service 3 | 3 | 12.00 | 0.90 | 11.10 | 10.00 |
| Service 1, Service 2, Service 3, Service 4 | 4 | 12.00 | 1.60 | 10.40 | 9.50 |
| Service 1, Service 2, Service 3, Service 4, Service 5 | 5 | 12.00 | 2.50 | 9.50 | 9.00 |
| Service 1, Service 2, Service 3, Service 4, Service 5, Service 6 | 6 | 12.00 | 3.60 | 8.40 | 8.50 |
| Service 1, Service 2, Service 3, Service 4, Service 5, Service 6, Service 7 | 7 | 12.00 | 4.90 | 7.10 | 8.00 |
| Service 1, Service 2, Service 3, Service 4, Service 5, Service 6, Service 7,Service 8 | 8 | 12.00 | 6.40 | 5.60 | 7.50 |
| Service 1, Service 2, Service 3, Service 4, Service 5, Service 6, Service 7,Service 8, Service 9 | 9 | 12.00 | 8.10 | 3.90 | 7.00 |
| Service 1, Service 2, Service 3, Service 4, Service 5, Service 6, Service 7,Service 8, Service 9, Service 10 | 10 | 12.00 | 10.00 | 2.00 | 6.50 |

Your Decision (Please fill in the box with the number of health care services you provide to the patient, must be an integer from 0 to 10)

|  |
| --- |

1. Patient's disease is A/severity is moderate

| **Medical services** | **quantity** | **Your Consultation Fees (token)** | **Your Costs (token)** | **Your Profits**  **(token)** | **Patient Benefit (token)** |
| --- | --- | --- | --- | --- | --- |
| None | 0 | 0.00 | 0.00 | 0.00 | 0.00 |
| Service 1 | 1 | 10.00 | 0.10 | 9.90 | 0.75 |
| Service 1, Service 2 | 2 | 10.00 | 0.40 | 9.60 | 1.50 |
| Service 1, Service 2, Service 3 | 3 | 10.00 | 0.90 | 9.10 | 2.00 |
| Service 1, Service 2, Service 3, Service 4 | 4 | 10.00 | 1.60 | 8.40 | 7.00 |
| Service 1, Service 2, Service 3, Service 4, Service 5 | 5 | 10.00 | 2.50 | 7.50 | 10.00 |
| Service 1, Service 2, Service 3, Service 4, Service 5, Service 6 | 6 | 10.00 | 3.60 | 6.40 | 9.50 |
| Service 1, Service 2, Service 3, Service 4, Service 5, Service 6, Service 7 | 7 | 10.00 | 4.90 | 5.10 | 9.00 |
| Service 1, Service 2, Service 3, Service 4, Service 5, Service 6, Service 7,Service 8 | 8 | 10.00 | 6.40 | 3.60 | 8.50 |
| Service 1, Service 2, Service 3, Service 4, Service 5, Service 6, Service 7,Service 8, Service 9 | 9 | 10.00 | 8.10 | 1.90 | 8.00 |
| Service 1, Service 2, Service 3, Service 4, Service 5, Service 6, Service 7,Service 8, Service 9, Service 10 | 10 | 10.00 | 10.00 | 0.00 | 7.50 |

Your Decision (Please fill in the box with the number of health care services you provide to the patient, must be an integer from 0 to 10)

|  |
| --- |

1. Patient's disease is B/severity is moderate

| **Medical services** | **quantity** | **Your Consultation Fees (token)** | **Your Costs (token)** | **Your Profits**  **(token)** | **Patient Benefit (token)** |
| --- | --- | --- | --- | --- | --- |
| None | 0 | 0.00 | 0.00 | 0.00 | 0.00 |
| Service 1 | 1 | 13.00 | 0.10 | 12.90 | 0.75 |
| Service 1, Service 2 | 2 | 13.00 | 0.40 | 12.60 | 1.50 |
| Service 1, Service 2, Service 3 | 3 | 13.00 | 0.90 | 12.10 | 2.00 |
| Service 1, Service 2, Service 3, Service 4 | 4 | 13.00 | 1.60 | 11.40 | 7.00 |
| Service 1, Service 2, Service 3, Service 4, Service 5 | 5 | 13.00 | 2.50 | 10.50 | 10.00 |
| Service 1, Service 2, Service 3, Service 4, Service 5, Service 6 | 6 | 13.00 | 3.60 | 9.40 | 9.50 |
| Service 1, Service 2, Service 3, Service 4, Service 5, Service 6, Service 7 | 7 | 13.00 | 4.90 | 8.10 | 9.00 |
| Service 1, Service 2, Service 3, Service 4, Service 5, Service 6, Service 7,Service 8 | 8 | 13.00 | 6.40 | 6.60 | 8.50 |
| Service 1, Service 2, Service 3, Service 4, Service 5, Service 6, Service 7,Service 8, Service 9 | 9 | 13.00 | 8.10 | 4.90 | 8.00 |
| Service 1, Service 2, Service 3, Service 4, Service 5, Service 6, Service 7,Service 8, Service 9, Service 10 | 10 | 13.00 | 10.00 | 3.00 | 7.50 |

Your Decision (Please fill in the box with the number of health care services you provide to the patient, must be an integer from 0 to 10)

|  |
| --- |

1. Patient's disease is C/severity is moderate

| **Medical services** | **quantity** | **Your Consultation Fees (token)** | **Your Costs (token)** | **Your Profits**  **(token)** | **Patient Benefit (token)** |
| --- | --- | --- | --- | --- | --- |
| None | 0 | 0.00 | 0.00 | 0.00 | 0.00 |
| Service 1 | 1 | 9.00 | 0.10 | 8.90 | 0.75 |
| Service 1, Service 2 | 2 | 9.00 | 0.40 | 8.60 | 1.50 |
| Service 1, Service 2, Service 3 | 3 | 9.00 | 0.90 | 8.10 | 2.00 |
| Service 1, Service 2, Service 3, Service 4 | 4 | 9.00 | 1.60 | 7.40 | 7.00 |
| Service 1, Service 2, Service 3, Service 4, Service 5 | 5 | 9.00 | 2.50 | 6.50 | 10.00 |
| Service 1, Service 2, Service 3, Service 4, Service 5, Service 6 | 6 | 9.00 | 3.60 | 5.40 | 9.50 |
| Service 1, Service 2, Service 3, Service 4, Service 5, Service 6, Service 7 | 7 | 9.00 | 4.90 | 4.10 | 9.00 |
| Service 1, Service 2, Service 3, Service 4, Service 5, Service 6, Service 7,Service 8 | 8 | 9.00 | 6.40 | 2.60 | 8.50 |
| Service 1, Service 2, Service 3, Service 4, Service 5, Service 6, Service 7,Service 8, Service 9 | 9 | 9.00 | 8.10 | 0.90 | 8.00 |
| Service 1, Service 2, Service 3, Service 4, Service 5, Service 6, Service 7,Service 8, Service 9, Service 10 | 10 | 9.00 | 10.00 | -1.00 | 7.50 |

Your Decision (Please fill in the box with the number of health care services you provide to the patient, must be an integer from 0 to 10)

|  |
| --- |

1. Patient's disease is D/severity is moderate

| **Medical services** | **quantity** | **Your Consultation Fees (token)** | **Your Costs (token)** | **Your Profits**  **(token)** | **Patient Benefit (token)** |
| --- | --- | --- | --- | --- | --- |
| None | 0 | 0.00 | 0.00 | 0.00 | 0.00 |
| Service 1 | 1 | 15.00 | 0.10 | 14.90 | 0.75 |
| Service 1, Service 2 | 2 | 15.00 | 0.40 | 14.60 | 1.50 |
| Service 1, Service 2, Service 3 | 3 | 15.00 | 0.90 | 14.10 | 2.00 |
| Service 1, Service 2, Service 3, Service 4 | 4 | 15.00 | 1.60 | 13.40 | 7.00 |
| Service 1, Service 2, Service 3, Service 4, Service 5 | 5 | 15.00 | 2.50 | 12.50 | 10.00 |
| Service 1, Service 2, Service 3, Service 4, Service 5, Service 6 | 6 | 15.00 | 3.60 | 11.40 | 9.50 |
| Service 1, Service 2, Service 3, Service 4, Service 5, Service 6, Service 7 | 7 | 15.00 | 4.90 | 10.10 | 9.00 |
| Service 1, Service 2, Service 3, Service 4, Service 5, Service 6, Service 7,Service 8 | 8 | 15.00 | 6.40 | 8.60 | 8.50 |
| Service 1, Service 2, Service 3, Service 4, Service 5, Service 6, Service 7,Service 8, Service 9 | 9 | 15.00 | 8.10 | 6.90 | 8.00 |
| Service 1, Service 2, Service 3, Service 4, Service 5, Service 6, Service 7,Service 8, Service 9, Service 10 | 10 | 15.00 | 10.00 | 5.00 | 7.50 |

Your Decision (Please fill in the box with the number of health care services you provide to the patient, must be an integer from 0 to 10)

|  |
| --- |

1. Patient's disease is E/severity is moderate

| **Medical services** | **quantity** | **Your Consultation Fees (token)** | **Your Costs (token)** | **Your Profits**  **(token)** | **Patient Benefit (token)** |
| --- | --- | --- | --- | --- | --- |
| None | 0 | 0.00 | 0.00 | 0.00 | 0.00 |
| Service 1 | 1 | 12.00 | 0.10 | 11.90 | 0.75 |
| Service 1, Service 2 | 2 | 12.00 | 0.40 | 11.60 | 1.50 |
| Service 1, Service 2, Service 3 | 3 | 12.00 | 0.90 | 11.10 | 2.00 |
| Service 1, Service 2, Service 3, Service 4 | 4 | 12.00 | 1.60 | 10.40 | 7.00 |
| Service 1, Service 2, Service 3, Service 4, Service 5 | 5 | 12.00 | 2.50 | 9.50 | 10.00 |
| Service 1, Service 2, Service 3, Service 4, Service 5, Service 6 | 6 | 12.00 | 3.60 | 8.40 | 9.50 |
| Service 1, Service 2, Service 3, Service 4, Service 5, Service 6, Service 7 | 7 | 12.00 | 4.90 | 7.10 | 9.00 |
| Service 1, Service 2, Service 3, Service 4, Service 5, Service 6, Service 7,Service 8 | 8 | 12.00 | 6.40 | 5.60 | 8.50 |
| Service 1, Service 2, Service 3, Service 4, Service 5, Service 6, Service 7,Service 8, Service 9 | 9 | 12.00 | 8.10 | 3.90 | 8.00 |
| Service 1, Service 2, Service 3, Service 4, Service 5, Service 6, Service 7,Service 8, Service 9, Service 10 | 10 | 12.00 | 10.00 | 2.00 | 7.50 |

Your Decision (Please fill in the box with the number of health care services you provide to the patient, must be an integer from 0 to 10)

|  |
| --- |

1. Patient's disease is A/severity is severe

| **Medical services** | **quantity** | **Your Consultation Fees (token)** | **Your Costs (token)** | **Your Profits**  **(token)** | **Patient Benefit (token)** |
| --- | --- | --- | --- | --- | --- |
| None | 0 | 0.00 | 0.00 | 0.00 | 0.00 |
| Service 1 | 1 | 10.00 | 0.10 | 9.90 | 0.75 |
| Service 1, Service 2 | 2 | 10.00 | 0.40 | 9.60 | 2.20 |
| Service 1, Service 2, Service 3 | 3 | 10.00 | 0.90 | 9.10 | 4.05 |
| Service 1, Service 2, Service 3, Service 4 | 4 | 10.00 | 1.60 | 8.40 | 6.00 |
| Service 1, Service 2, Service 3, Service 4, Service 5 | 5 | 10.00 | 2.50 | 7.50 | 7.75 |
| Service 1, Service 2, Service 3, Service 4, Service 5, Service 6 | 6 | 10.00 | 3.60 | 6.40 | 9.00 |
| Service 1, Service 2, Service 3, Service 4, Service 5, Service 6, Service 7 | 7 | 10.00 | 4.90 | 5.10 | 9.45 |
| Service 1, Service 2, Service 3, Service 4, Service 5, Service 6, Service 7,Service 8 | 8 | 10.00 | 6.40 | 3.60 | 8.80 |
| Service 1, Service 2, Service 3, Service 4, Service 5, Service 6, Service 7,Service 8, Service 9 | 9 | 10.00 | 8.10 | 1.90 | 6.75 |
| Service 1, Service 2, Service 3, Service 4, Service 5, Service 6, Service 7,Service 8, Service 9, Service 10 | 10 | 10.00 | 10.00 | 0.00 | 3.00 |

Your Decision (Please fill in the box with the number of health care services you provide to the patient, must be an integer from 0 to 10)

|  |
| --- |

1. Patient's disease is B/severity is severe

| **Medical services** | **quantity** | **Your Consultation Fees (token)** | **Your Costs (token)** | **Your Profits**  **(token)** | **Patient Benefit (token)** |
| --- | --- | --- | --- | --- | --- |
| None | 0 | 0.00 | 0.00 | 0.00 | 0.00 |
| Service 1 | 1 | 13.00 | 0.10 | 12.90 | 0.75 |
| Service 1, Service 2 | 2 | 13.00 | 0.40 | 12.60 | 2.20 |
| Service 1, Service 2, Service 3 | 3 | 13.00 | 0.90 | 12.10 | 4.05 |
| Service 1, Service 2, Service 3, Service 4 | 4 | 13.00 | 1.60 | 11.40 | 6.00 |
| Service 1, Service 2, Service 3, Service 4, Service 5 | 5 | 13.00 | 2.50 | 10.50 | 7.75 |
| Service 1, Service 2, Service 3, Service 4, Service 5, Service 6 | 6 | 13.00 | 3.60 | 9.40 | 9.00 |
| Service 1, Service 2, Service 3, Service 4, Service 5, Service 6, Service 7 | 7 | 13.00 | 4.90 | 8.10 | 9.45 |
| Service 1, Service 2, Service 3, Service 4, Service 5, Service 6, Service 7,Service 8 | 8 | 13.00 | 6.40 | 6.60 | 8.80 |
| Service 1, Service 2, Service 3, Service 4, Service 5, Service 6, Service 7,Service 8, Service 9 | 9 | 13.00 | 8.10 | 4.90 | 6.75 |
| Service 1, Service 2, Service 3, Service 4, Service 5, Service 6, Service 7,Service 8, Service 9, Service 10 | 10 | 13.00 | 10.00 | 3.00 | 3.00 |

Your Decision (Please fill in the box with the number of health care services you provide to the patient, must be an integer from 0 to 10)

|  |
| --- |

1. Patient's disease is C/severity is severe

| **Medical services** | **quantity** | **Your Consultation Fees (token)** | **Your Costs (token)** | **Your Profits**  **(token)** | **Patient Benefit (token)** |
| --- | --- | --- | --- | --- | --- |
| None | 0 | 0.00 | 0.00 | 0.00 | 0.00 |
| Service 1 | 1 | 9.00 | 0.10 | 8.90 | 0.75 |
| Service 1, Service 2 | 2 | 9.00 | 0.40 | 8.60 | 2.20 |
| Service 1, Service 2, Service 3 | 3 | 9.00 | 0.90 | 8.10 | 4.05 |
| Service 1, Service 2, Service 3, Service 4 | 4 | 9.00 | 1.60 | 7.40 | 6.00 |
| Service 1, Service 2, Service 3, Service 4, Service 5 | 5 | 9.00 | 2.50 | 6.50 | 7.75 |
| Service 1, Service 2, Service 3, Service 4, Service 5, Service 6 | 6 | 9.00 | 3.60 | 5.40 | 9.00 |
| Service 1, Service 2, Service 3, Service 4, Service 5, Service 6, Service 7 | 7 | 9.00 | 4.90 | 4.10 | 9.45 |
| Service 1, Service 2, Service 3, Service 4, Service 5, Service 6, Service 7,Service 8 | 8 | 9.00 | 6.40 | 2.60 | 8.80 |
| Service 1, Service 2, Service 3, Service 4, Service 5, Service 6, Service 7,Service 8, Service 9 | 9 | 9.00 | 8.10 | 0.90 | 6.75 |
| Service 1, Service 2, Service 3, Service 4, Service 5, Service 6, Service 7,Service 8, Service 9, Service 10 | 10 | 9.00 | 10.00 | -1.00 | 3.00 |

Your Decision (Please fill in the box with the number of health care services you provide to the patient, must be an integer from 0 to 10)

|  |
| --- |

1. Patient's disease is D/severity is severe

| **Medical services** | **quantity** | **Your Consultation Fees (token)** | **Your Costs (token)** | **Your Profits**  **(token)** | **Patient Benefit (token)** |
| --- | --- | --- | --- | --- | --- |
| None | 0 | 0.00 | 0.00 | 0.00 | 0.00 |
| Service 1 | 1 | 15.00 | 0.10 | 14.90 | 0.75 |
| Service 1, Service 2 | 2 | 15.00 | 0.40 | 14.60 | 2.20 |
| Service 1, Service 2, Service 3 | 3 | 15.00 | 0.90 | 14.10 | 4.05 |
| Service 1, Service 2, Service 3, Service 4 | 4 | 15.00 | 1.60 | 13.40 | 6.00 |
| Service 1, Service 2, Service 3, Service 4, Service 5 | 5 | 15.00 | 2.50 | 12.50 | 7.75 |
| Service 1, Service 2, Service 3, Service 4, Service 5, Service 6 | 6 | 15.00 | 3.60 | 11.40 | 9.00 |
| Service 1, Service 2, Service 3, Service 4, Service 5, Service 6, Service 7 | 7 | 15.00 | 4.90 | 10.10 | 9.45 |
| Service 1, Service 2, Service 3, Service 4, Service 5, Service 6, Service 7,Service 8 | 8 | 15.00 | 6.40 | 8.60 | 8.80 |
| Service 1, Service 2, Service 3, Service 4, Service 5, Service 6, Service 7,Service 8, Service 9 | 9 | 15.00 | 8.10 | 6.90 | 6.75 |
| Service 1, Service 2, Service 3, Service 4, Service 5, Service 6, Service 7,Service 8, Service 9, Service 10 | 10 | 15.00 | 10.00 | 5.00 | 3.00 |

Your Decision (Please fill in the box with the number of health care services you provide to the patient, must be an integer from 0 to 10)

|  |
| --- |

1. Patient's disease is E/severity is severe

| **Medical services** | **quantity** | **Your Consultation Fees (token)** | **Your Costs (token)** | **Your Profits**  **(token)** | **Patient Benefit (token)** |
| --- | --- | --- | --- | --- | --- |
| None | 0 | 0.00 | 0.00 | 0.00 | 0.00 |
| Service 1 | 1 | 12.00 | 0.10 | 11.90 | 0.75 |
| Service 1, Service 2 | 2 | 12.00 | 0.40 | 11.60 | 2.20 |
| Service 1, Service 2, Service 3 | 3 | 12.00 | 0.90 | 11.10 | 4.05 |
| Service 1, Service 2, Service 3, Service 4 | 4 | 12.00 | 1.60 | 10.40 | 6.00 |
| Service 1, Service 2, Service 3, Service 4, Service 5 | 5 | 12.00 | 2.50 | 9.50 | 7.75 |
| Service 1, Service 2, Service 3, Service 4, Service 5, Service 6 | 6 | 12.00 | 3.60 | 8.40 | 9.00 |
| Service 1, Service 2, Service 3, Service 4, Service 5, Service 6, Service 7 | 7 | 12.00 | 4.90 | 7.10 | 9.45 |
| Service 1, Service 2, Service 3, Service 4, Service 5, Service 6, Service 7,Service 8 | 8 | 12.00 | 6.40 | 5.60 | 8.80 |
| Service 1, Service 2, Service 3, Service 4, Service 5, Service 6, Service 7,Service 8, Service 9 | 9 | 12.00 | 8.10 | 3.90 | 6.75 |
| Service 1, Service 2, Service 3, Service 4, Service 5, Service 6, Service 7,Service 8, Service 9, Service 10 | 10 | 12.00 | 10.00 | 2.00 | 3.00 |

Your Decision (Please fill in the box with the number of health care services you provide to the patient, must be an integer from 0 to 10)

|  |
| --- |
